# Correlation of *T*_max_ volumes with clinical outcome in anterior circulation stroke

**DOI:** 10.1002/brb3.772

**Published:** 2017-07-26

**Authors:** Fatih Seker, Johannes Pfaff, Arne Potreck, Sibu Mundiyanapurath, Peter A. Ringleb, Martin Bendszus, Markus A. Möhlenbruch

**Affiliations:** ^1^ Department of Neuroradiology Heidelberg University Hospital Heidelberg Germany; ^2^ Department of Neurology Heidelberg University Hospital Heidelberg Germany

**Keywords:** outcome, perfusion, stroke, thrombectomy, *T*_max_

## Abstract

**Background and Purpose:**

The recent thrombectomy trials have shown that perfusion imaging is helpful in proper patient selection in thromboembolic stroke. In this study, we analyzed the correlation of pretreatment *T*_max_ volumes in MR and CT perfusion with clinical outcome after thrombectomy.

**Methods:**

Forty‐one consecutive patients with middle cerebral artery occlusion (MCA) or carotid T occlusion treated with thrombectomy were included. *T*_max_ volumes at delays of >4, 6, 8, and 10 s as well as infarct core and mismatch ratio were automatically estimated in preinterventional MRI or CT perfusion using RAPID software. These perfusion parameters were correlated with clinical outcome. Outcome was assessed using modified Rankin scale at 90 days.

**Results:**

In patients with successful recanalization of MCA occlusion, *T*_max_ > 8 and 10 s showed the best linear correlation with clinical outcome (*r* = 0.75; *p* = .0139 and *r* = 0.73; *p* = .0139), better than infarct core (*r* = 0.43; *p* = .2592). In terminal internal carotid artery occlusions, none of the perfusion parameters showed a significant correlation with clinical outcome.

**Conclusions:**

*T*_max_ at delays of >8 and 10 s is a good predictor for clinical outcome in MCA occlusions. In carotid T occlusion, however, *T*_max_ volumes do not correlate with outcome.

## 1 INTRODUCTION

MR and CT perfusion are successfully used in clinical routine for the estimation of salvageable tissue in acute ischemic stroke, which can be helpful in identifying patients eligible for mechanical thrombectomy (Butcher et al., 2005; Campbell et al., 2012, 2015). Often time to peak (TTP) or *T*_max_ are used for the estimation of hypoperfused volume (Lansberg et al., 2012). Compared to TTP parameter, *T*_max_ has the advantage of a reduced dependence on bolus shape and cardiac output (Olivot et al., 2009; Straka, Albers, & Bammer, 2010). Also, *T*_max_ values in MR and CT perfusion have been shown to be comparable (Campbell et al., 2012; Lin, Bivard, Levi, & Parsons, 2014).

*T*_max_ volumes with different delay thresholds, infarct core, and mismatch ratio can be estimated automatically using analysis software such as RAPID (Austein et al., 2016; Dehkharghani et al., 2015; Lansberg et al., 2011). These estimations are based on diffusion‐weighted images (DWI) and deconvolution of dynamic susceptibility contrast perfusion (DSC‐PWI) in MRI or volume perfusion CT.

Previous studies have shown that infarct core correlates well with collaterals and final infarct volume in anterior circulation stroke (Lee et al., 2015; Mokin et al., 2017; Seker, Potreck, Möhlenbruch, Bendszus, & Pham, 2016). The correlation of various *T*_max_ volumes with clinical outcome, however, is not clear yet. Therefore, we analyzed the correlation of *T*_max_ volumes, infarct core, and mismatch ratio with clinical outcome after thrombectomy in anterior circulation stroke.

## 2 METHODS

### 2.1 Patients

Between March 2014 and April 2015 all consecutive patients undergoing neurothrombectomy for the treatment of acute ischemic stroke were collected in a prospective database and analyzed retrospectively. Inclusion criteria for this study were an acute occlusion of the terminal carotid internal artery (carotid T) or the M1 segment of the middle cerebral artery (MCA), premorbid modified Rankin scale score of ≤2, and treatment with thrombectomy. In total, 41 patients met the inclusion criteria for this retrospective study. Baseline demographic data, initial National Institutes of Health Stroke Scale (NIHSS) score, and modified Rankin scale score at 90 days after stroke onset (90d‐mRS) were regularly documented by a neurologist. Good neurological outcome was defined as 90d‐mRS score of ≤2 and poor outcome was defined as 90d‐mRS >2. Our institutional review board approved the study.

### 2.2 Imaging protocol

All patients underwent either multimodal CT imaging or MRI prior to thrombectomy as described recently (Potreck et al., 2017; Seker et al., 2016).

Briefly, multimodal CT imaging was performed using a 64‐multislice CT (SOMATOM Definition AS, Siemens, Erlangen, Germany). Scanning order was nonenhanced CT, volume perfusion CT, and single‐phase CT angiography. Acquisition parameters for CT perfusion were 180 kV and 80 mAs, acquisition duration was 60 s. A single contrast bolus of 36 ml Xenetix 350 (Guerbet, Sulzbach, Germany) was applied at a flow rate of 6.0 ml/s, followed by a saline flush of 20 ml and a flow rate of 6.0 ml/s. CT perfusion was reconstructed with a slice thickness of 5 mm every 3 mm. Acquisition parameters for CTA were 120 kV and 20 mAs. A single contrast bolus of 65 ml Xenetix 350 was given with a flow rate of 4.0 ml/s, followed by a saline flush of 20 ml and a flow rate of 4.0 ml/s (Seker et al., 2016).

MR images were acquired using two MRI scanners with a field strength of 3Tesla (Magnetom Trio TIM and Verio, Siemens Healthcare, Erlangen, Germany). The MRI protocol included DWI, susceptibility‐weighted imaging, FLAIR, T2 turbo spin echo, time‐of‐flight angiography, contrast‐enhanced MR angiography and DSC‐PWI. DWI was performed using a single‐shot spin‐echo echo‐planar sequence with repetition time and echo time (TR/TE) = 5,300 ms/90 ms, a flip angle of 90° and a slice thickness (ST) of 5 mm. Diffusion sensitizing gradients were applied with *b* = 0 and *b* = 1,200 s/mm^2^. 2D‐T2‐TSE‐weighted and 2D‐FLAIR‐weighted images were acquired with TR=5,000 ms/8,500 ms, TE=85 ms/133 ms, ST = 5 mm, and slice gap = 0.5 mm. DSC‐PWI was performed with a T2*‐weighted gradient‐echo echo‐planar imaging sequence and was started with bolus injection of a 0.1 mmol/kg of intravenous gadoterate meglumine. Bolus and prebolus were injected at an injection rate of 5 ml/s. Sections (25–27) were imaged with fat suppression (TR/TE = 2220 ms/36 ms, flip angle: 90°, field of view: 240 × 240 mm, image matrix: 128 × 128 mm, ST=5 mm). In total, 50–75 dynamic measurements were performed (including at least eight prebolus measurements) (Potreck et al., 2017).

### 2.3 Image analysis

Recanalization success was assessed on final angiograms. Successful recanalization was defined as Thrombolysis In Cerebral Infarction (TICI) score of 2b‐3.

### 2.4 Postprocessing

Preinterventional DWI and DSC‐PWI or volume perfusion CT data were processed by RAPID software (research version, Stanford University). RAPID automatically identified infarct core, mismatch ratio, and volumes at four *T*_max_ thresholds (delay of >4 s, >6 s, >8 s, and >10 s).

Estimation of ischemic core was based on either relative CBF (threshold of <31% of mean contralateral CBF) or apparent diffusion coefficient maps (Campbell et al., 2011; Straka et al., 2010). Mismatch was defined as perfusion lesion volume (*T*_max_ delay of >6 s) to infarct core (diffusion lesion or CT‐relative hemispheric cerebral blood flow decrease <30%) volume ratio >1.2 with >10 ml absolute mismatch and ischemic core volume <70 ml (Campbell et al., 2015).

### 2.5 Statistical analysis

Statistical analyses were performed using R version 3.2.2 and RStudio version 0.99. *T*_max_ volumes at thresholds of 4, 6, 8, and 10 s, infarct core volume and mismatch ratio were correlated with 90d‐mRS using Pearson's correlation. *T* test was used for nonparametric univariate analyses. Statistical significance was set at *p* < .05. Given multiple comparisons, Benjamini–Hochberg correction was used to minimize the false‐discovery rate.

## 3 RESULTS

### 3.1 Patient characteristics

The demographic characteristics of the patients are shown in Table 1.

**Table 1:** Patient characteristics

Characteristics	Total (*n* = 41)	MCA (*n* = 24)	Carotid T (*n* = 17)
Age (years), mean ± SD	70.2 ± 11.4	70.2 ± 12.0	70.2 ± 10.8
Female, *n* (%)	19 (46.3)	13 (54.1)	6 (35.2)
Hypertension, *n* (%)	29 (70.7)	17 (70.8)	12 (70.5)
Cardiac arrhythmia, *n* (%)	14 (34.1)	9 (37.5)	5 (29.4)
Coronary heart disease, *n* (%)	9 (21.9)	5 (20.8)	4 (23.5)
Diabetes, *n* (%)	8 (19.5)	6 (25.0)	2 (11.7)
Prestroke mRS, median (IQR)	0 (0–1)	0 (0–1)	0 (0–0)
Initial NIHSS score, median(IQR)	19 (15–21)	16 (13.5–20.5)	20 (18–22)
Imaging modality			
CT, *n* (%)	24 (58.5)	12 (50.0)	12 (70.6)
MRI, *n* (%)	17 (41.4)	12 (50.0)	5 (29.4)
Intravenous tPA, *n* (%)	31 (75.6)	18 (75.0)	13 (76.4)
Onset to imaging (min),median (IQR)	94 (68–143)	95 (68–140)	94 (65–139)
Onset to TICI (min), median(IQR)	279 (225–336)	270 (235–296)	279 (222–372)
Recanalization successful			
TICI 0‐2a, *n* (%)	9 (21.9)	5 (20.8)	4 (23.5)
TICI 2b‐3, *n* (%)	32 (78.0)	19 (79.2)	13 (76.5)
90d‐mRS, median (IQR)	3 (1.75–4)	3 (1–4)	3 (2–4)

### 3.2 MCA occlusion

**Figure 1:**
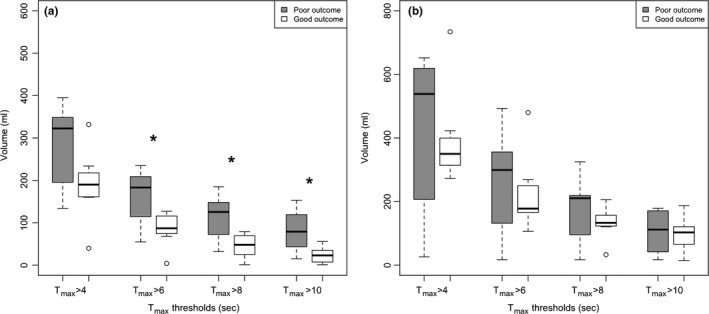
(a) In middle cerebral artery occlusion, *T*_max_ volumes at delays of 6, 8, and 10 s in patients with poor outcome were significantly higher compared to patients with good outcome (*p* = .1350, *p* = .0157, *p* = .0139, and *p* = .0146, respectively). (b) In carotid T occlusion, there were no significant differences (*p* = .9743, *p* = .7805, *p* = .2593, and *p* = .4216). The asterisks indicate statistically significant differences (*p *<* *.05)

In MCA occlusion, *T*_max_ volumes at a delay of >8 and 10 s showed the best linear correlation with clinical outcome (*r* = 0.68; *p* = .0139 and *r* = 0.67; *p* = .0139), better than infarct core (*r* = 0.51; *p* = .5299). *T*_max_ at >8 and 10 s did not show a significant correlation with infarct core (*r* = 0.10; *p* = .6581 and *r* = 0.19; *p* = .3678). Linear correlation was even better in patients with successful recanalization (*T*_max_ at >8 s, *r* = 0.75; *p* = .0139 and *T*_max_ at >10 s, *r* = 0.73; *p* = .0139) (Table 2). Furthermore, *T*_max_ volumes at >6, 8, and 10 s were significantly higher in patients with poor outcome compared to good outcome (Figure 1a).

**Table 2:** Correlation of perfusion parameters and clinical outcome

	90d‐mRS in MCA occlusion		90d‐mRS in carotid T occlusion	
	Total (*n* = 24)	TICI 2b‐3 (*n* = 19)	Total (*n* = 17)	TICI 2b‐3 (*n* = 13)
Infarct core	*r* = .51; *p* = .5299	*r* = .43; *p* = .2592	*r* = .11; *p* = .8257	*r* = −.07; *p* = .9060
Mismatch ratio	*r* = −.21; *p* = .7373	*r* = −.17; *p* = .7805	*r* = −.02; *p* = .9596	*r* = −.17; *p* = .7805
*T*_max_ > 4 s	*r* = .33; *p* = .3830	*r* = .57; *p* = .0875	*r* = .02; *p* = .9596	*r* = .18; *p* = .7805
*T*_max_ > 6 s	*r* = .61; *p* = .0198	*r* = .72; *p* = .0146	*r* = .09; *p* = .8519	*r* = .16; *p* = .7805
*T*_max_ > 8 s	*r* = .68; *p* = .0139	*r* = .75; *p* = .0139	*r* = .19; *p* = .7805	*r* = .17; *p* = .7805
*T*_max_ > 10 s	*r* = .67; *p* = .0139	*r* = .73; *p* = .0139	*r* = .20; *p* = .7588	*r* = .10; *p* = .8519

### 3.3 Carotid T occlusion

In terminal internal carotid artery occlusion, none of the perfusion parameters including infarct core and mismatch ratio showed a significant correlation with clinical outcome (Table 2). Also, no significant differences in *T*_max_ volumes were determined between patients with good and poor clinical outcomes (Figure 1b).

## 4 DISCUSSION

The past thrombectomy trials have shown that proper patient selection is important in mechanical thrombectomy (Meckel & Herweh, 2015; Sheth et al., 2016). In EXTEND‐IA and SWIFT PRIME, MR and CT perfusion were successfully used for patient selection (Campbell et al., 2015; Saver et al., 2015). Both MR and CT perfusion help distinguishing between infarct core and salvageable tissue. In EXTEND‐IA, for instance, *T*_max_ at a delay of >6 s was defined as hypoperfused volume and either cerebral blood flow (CBF) in CT or DWI in MRI were used to determine infarct core volume.

Unfortunately, there are differences in perfusion maps generated by the software which are provided by the manufacturers (Kudo et al., 2009). RAPID, however, allows an automatic postprocessing of MRI and CT perfusion and provides volumes for specific *T*_max_ perfusion delays as well as infarct core volume and mismatch ratio in a user‐friendly way which is currently not offered by manufacturers (Dehkharghani et al., 2015).

Various perfusion parameters in both CT and MRI have been studied (Lansberg et al., 2011; Mundiyanapurath et al., 2017; Seker et al., 2016; Straka et al., 2010). Mokin et al. (2017) and Albers et al. (2016) showed that CBF and cerebral blood volume (CBV) provide accurate prediction of final infarct volume after successful recanalization. Past studies have also demonstrated that *T*_max_ volume at a delay of >6 and/or 8 s accurately differentiate penumbra from salvageable tissue (Shih et al., 2003; Zhang et al., 2015). The correlation of *T*_max_ volumes with clinical outcome is not clear yet, though. We, therefore, performed a retrospective analysis on the correlation of *T*_max_ values at perfusion delays of >4, 6, 8, and 10 s with clinical outcome in anterior circulation stroke using RAPID.

In patients with successful recanalization of MCA occlusion, *T*_max_ volumes at thresholds of 8 and 10 s showed good linear correlation with clinical outcome with significant results (*r* = 0.75; *p* = .0139 and *r* = 0.73; *p* = .0139), even better than infarct core (*r* = 0.43; *p* = .2592) which is based on CBF or DWI in RAPID algorithm (Table 2). The underlying reasons are not clear. It could be analyzed, for instance, whether there is also a correlation of *T*_max_ ASPECTS (at a delay of >8 or 10 s) with clinical outcome (Tsogkas et al., 2016).

Unlike MCA occlusion, none of the *T*_max_ volumes had a significant correlation with clinical outcome in terminal internal carotid artery occlusions. Perhaps due to reduced flow within the ipsilateral anterior cerebral artery and posterior communicating artery, cerebral hemodynamics in carotid T occlusion differ from MCA occlusion which may result in weaker correlation of *T*_max_ volumes with clinical outcome. However, there are no studies confirming this hypothesis, yet.

RAPID was available only for a limited period at our institution. This explains why only 41 patients were included in this retrospective study. The low number of patients certainly is an important limitation of this study and further studies with larger study populations are necessary to confirm our findings. We, therefore, refrain from relying solely on *T*_max_ for outcome predictions. Besides, collaterals were not assessed in this study, which might have confounding effects. Preinterventional imaging in our study was not consistent, because 24 patients underwent CT imaging, while 17 patients underwent MRI using two MRI scanners with field strength of 3Tesla, which is also a limitation. However, RAPID has been shown to be a reliable tool in both CT and MR perfusion with comparable results (Austein et al., 2016; Campbell et al., 2012; Dehkharghani et al., 2015; Straka et al., 2010). Finally, patients who were not treated with endovascular therapy were not included in this analysis which is a potential source of selection bias.

## 5 CONCLUSIONS

*T*_max_ volumes at a delay of >8 and 10 s have a strong correlation with clinical outcome in middle cerebral artery occlusions as measured by RAPID. In terminal internal carotid artery occlusion, however, *T*_max_ volumes do not correlate with outcome. Further studies with larger study populations are necessary to confirm our findings, though.

## CONFLICT OF INTEREST

No conflicts of interest.
